# National Association of Dental Examiners

**Published:** 1890-09

**Authors:** 


### NATIONAL ASSOCIATION OF DENTAL EXAMINERS.

THE Ninth Annual Meeting of the National Association of Dental Examiners was held at Excelsior Springs, Mo., commencing Monday, August 4, 1890.

The following State Boards were represented:

*Colorado*, Dr. P. T. Smith.

*Illinois*, Dr. C. R. E. Koch.

*Iowa*, Drs. S. A. Garber, E. E. Hughes, and E. D. Brower.

*Pennsylvania*, Dr. Louis Jack.

*Maryland*, Dr. T. S. Waters.

*Kansas*, Drs. L. C. Wasson and A. M. Callahan.

*Ohio*, Drs. J. Taft and H. A. Smith.

*Minnesota*, Dr. J. H. Martindale.

During the sessions the Board of Registration in Dentistry for the State of Rhode Island and Providence Plantations, represented by Dr. Wm. P. Church, was elected to membership.

In the absence of the secretary, Dr. F. A. Levy, Dr. J. H. Martindale, of Minnesota, was elected secretary *pro tem.*

After discussion, the following resolution, offered by Dr. Jack and amended by Dr. Koch, was adopted, on motion of Dr. Taft:

"*Resolved*, That this body recommends the various examining boards under no circumstances to grant temporary licenses to dental students at any period of their course of instruction, whenever their State laws will permit them so to do."

Drs. Jack, Garber, and P. T. Smith were appointed a committee to formulate the principles which this Association would recommend should be incorporated in the State laws. This committee subsequently presented a report which, as amended and adopted, recommended the following principles for incorporation in laws for the regulation of dental practice or for the guidance of those framing them:

1. The creation of Boards of Examiners in each State.

2. The Boards to be officially created by the constituted appointing power of the various States, the appointees to be selected from a number of names presented by the representative State societies, each State society at its annual meeting placing in nomination not more than two names for each appointment to be made.

3. Recognizing five years' actual practice at the time of the passage of the law as qualifying for the continuance of practice.

4. Empowering the Board of Examiners to examine and grant certificates to non-graduates, provided the candidates present satisfactory evidence of having had at least five calendar years of instruction.

5. These and all other examinations to be both oral and written, and candidates to be also subject to tests of practical skill.

6. Empowering the Boards to examine graduates in dentistry.

7. Prohibiting medical graduates without special qualifications practising dentistry.

8. Requiring medical graduates to have their special qualifications determined by the same tests as other non-graduates in dentistry (see No. 5).

9. Making failure to pass the required examination in any one branch sufficient cause for refusal to grant the certificate.

10. Making failure in the practical tests in either of the two general departments of dentistry work disqualification.

11. Expressing the opinion that examinations for the special degree in dentistry should be conducted by a Board of Examiners established by law in each State, instead of by Faculties as at present; and the belief that the power to grant degrees must at length become vested in Boards created for the purpose.

12. Conferring on State Boards the power to revoke, for cause, a certificate of qualification previously granted.

The secretary was directed to call the attention of the American Dental Association to the fact that a case involving the constitutionality of the law regulating the practice of dentistry in New Hampshire is now pending in the Supreme Court of the United States, and asking them to see to it that it does not go by default.

Dr. Koch, from the committee on dental colleges, reported the following schools, the diplomas of which this Association recommends that the State Boards indorse:

American College of Dental Surgery, Chicago, Ill.

Baltimore College of Dental Surgery, Baltimore, Md.

Boston Dental College, Boston, Mass.

Chicago College of Dental Surgery, Chicago, Ill.

College of Dentistry, Department of Medicine, University of Minnesota, Minneapolis, Minn.

Dental Department, Columbian University, Washington, D. C.

Dental Department of Northwestern University, Chicago, Ill. (Now University Dental College.)

Dental Department of Southern Medical College, Atlanta, Ga.

Dental Department, University of Tennessee, Nashville, Tenn.

Harvard University, Dental Department, Cambridge, Mass.

Indiana Dental College, Indianapolis, Ind.

Kansas City Dental College, Kansas City, Mo.

Louisville College of Dentistry, Louisville, Ky.

Minnesota Hospital College, Dental Department, Minneapolis, Minn. (Defunct.)

Missouri Dental College, St. Louis, Mo.

New York College of Dentistry, New York, N. Y.

Ohio College of Dental Surgery, Cincinnati, O.

Pennsylvania College of Dental Surgery, Philadelphia, Pa.

Philadelphia Dental College, Philadelphia, Pa.

School of Dentistry of Meharry Medical Department of Central Tennessee College, Nashville, Tenn.

St. Paul Medical College, Dental Department, St. Paul, Minn. (Defunct.)

University of California, Dental Department, San Francisco, Cal.

Northwestern College of Dental Surgery, Chicago, Ill. (The diplomas of this college are discredited after 1889.)

State University of Iowa, Dental Department, Iowa City, Ia.

University of Maryland, Dental Department, Baltimore, Md.

University of Michigan, Dental Department, Ann Arbor, Mich.

University of Pennsylvania, Dental Department, Philadelphia, Pa.

Vanderbilt University, Dental Department, Nashville, Tenn.

The following officers were elected for the ensuing year: C. R. E. Koch, Chicago, Ill., President; L. C. Wasson, Topeka, Kan., Vice-President; J. H. Martindale, Minneapolis, Minn., Secretary and Treasurer. The president appointed as the Committee on Dental Colleges, Drs. Louis Jack, T. S. Waters, E. E. Hughes, W. P. Church, and J. H. Martindale.

On motion, the following committee was appointed to consider the advisability of holding the meetings at some other time and place than the annual meetings of the American Dental Association, with discretionary power in the matter: Drs. J. Taft, F. A. Levy, and S. A. Garber.

Adjourned to meet at the call of the president.

